# Constructal Optimization for Cooling a Non-Uniform Heat Generating Radial-Pattern Disc by Conduction

**DOI:** 10.3390/e20090685

**Published:** 2018-09-07

**Authors:** Jiang You, Huijun Feng, Lingen Chen, Zhihui Xie

**Affiliations:** 1 Institute of Thermal Science and Power Engineering, Naval University of Engineering, Wuhan 430033, China; 2 Military Key Laboratory for Naval Ship Power Engineering, Naval University of Engineering, Wuhan 430033, China; 3 College of Power Engineering, Naval University of Engineering, Wuhan 430033, China

**Keywords:** constructal theory, radial-pattern disc, non-uniform heat generation, minimum maximum temperature difference, variable cross-section, generalized thermodynamic optimization

## Abstract

A heat conduction model in a radial-pattern disc by considering non-uniform heat generation (NUHG) is established in this paper. A series of high conductivity channels (HCCs) are attached on the rim of the disc and extended to its center. Constructal optimizations of the discs with constant and variable cross-sectional HCCs are carried out, respectively, and their maximum temperature differences (MTDs) are minimized based on analytical method and finite element method. Besides, the influences of the NUHG coefficient, HCC number and width coefficient on the optimal results are studied. The results indicate that the deviation of the optimal constructs obtained from the analytical method and finite element method are comparatively slight. When the NUHG coefficient is equal to 10, the minimum MTD of the disc with 25 constant cross-sectional HCCs is specifically reduced by 48.8% compared to that with 10 HCCs. As a result, the heat conduction performance (HCP) of the disc can be efficiently improved by properly increasing the number of HCCs. The minimum MTD of the disc with variable cross-sectional HCC is decreased by 15.0% when the width coefficient is changed from 1 to 4. Therefore, the geometry of variable cross-sectional HCC can be applied in the constructal design of the disc to a better heat transfer performance. The constructal results obtained by investigating the non-uniform heat generating case in this paper can contribute to the design of practical electronic device to a better heat transfer performance.

## 1. Introduction

Nowadays, with the fast-developing electronic science and technology, the electronic components are faced with new trends of miniaturization, low power consumption, compound design and modularization, which are unprecedented opportunities and tremendous challenges for the electronic industry. As to miniature electronic components, the conventional heat transfer approaches such as convection or radiation are inevitably impossible to realize due to the space constraints. Accordingly, heat conduction has naturally been the prospective strategy for the effective cooling of electronic devices, and one way to enhance heat conduction is arranging high conductivity material. How to properly arrange the high conductivity material to reduce the hot spot temperature of the heat generating area is a hot topic in the context of heat conduction optimization [1,2,3].

Constructal theory [4,5,6] has emerged as the evolutionary design philosophy for finite size flow system evolution through time and it states that this evolution is based on a simple law: “for a finite size flow system to survive in time, it should evolve in such a way that it provides easier access to the flow that goes through it”. Invoking this law in the engineering field, one should bear in mind that this law is about the direction of evolution in time, and the design phenomenon is dynamic. Furthermore, the connotation of the time arrow design evolution has become the focus of concern by many researches and scholars [7,8,9,10,11,12,13,14,15,16,17,18,19,20,21,22,23,24,25,26]. Bejan [27] firstly conducted constructal optimization of a rectangular heat generating volume with the optimization objective of maximum temperature difference (MTD) and obtained the optimal distribution of constant high conductivity channels (HCCs). Neagu and Bejan [28,29] introduced a new geometric feature of variable cross-sectional high conductivity channels into the constructal method with the aim of further minimizing the thermal resistance. Thereafter, many scholars performed constructal optimizations of various heat generating bodies to improve their heat conduction performance (HCP), such as rectangular bodies [30,31,32,33], square bodies [34,35,36,37,38,39,40,41], triangular bodies [42,43,44,45], disc-shaped bodies with radial-pattern [46,47] and tree-shaped HCCs [48,49,50,51,52,53,54], respectively.

In the constructal designs of disc-shaped bodies, da Silva et al. [46] proposed a new heat conduction model in a radial-pattern disc in which the rectangular HCCs were mounted on the perimeter and protruded into the center. By taking the MTD as optimization objective, the constructal optimizations were carried out by analytical and numerical methods, respectively. The results showed that the number of HCCs and conductivity ratio had great effects on the optimal constructs of the disc. Sharifi et al. [47] carried out an optimization for the disc inserted with incomplete variable cross-sectional HCCs. The relevant results reported that the incomplete variable cross-sectional HCCs structure could further reduce the thermal resistance of the disc. In addition to the researches of radial-pattern disc, tree-shaped disc has been widely studied in the meantime. Rocha et al. [48] established a uniform heat generating model in a disc, and obtained the optimal arrangements of tree-shaped HCCs with the objective of MTD. Rocha et al. [49] further verified the results obtained in Ref. [46] by numerical calculations, and optimized the disc which included tree-shaped HCCs with loops. Xiao et al. [50] re-optimized the tree-shaped disc by releasing the constraint that the optimized last order constructs constituted the new order construct, and significantly reduced the MTD by 49.3%. Bahadormanesh and Salimpour [51] studied the disc-shaped body inserted with high-emissivity inserts, and obtained the best configurations of the disc by analytical and numerical methods, respectively. Sharfi et al. [52] investigated a solid disc using incomplete inserts of high thermal conductivity with the objective of thermal resistance minimization. Furthermore, the constructal studies of the heat conduction discs were extended from conventional scale to micro and nano scales. Chen et al. [53] re-optimized the disc-shaped body under the circumstances of micro and nano scales, and the results indicated that the constructs with size effects could improve the HCP with authority. Daneshi et al. [54] numerically calculated the disc-shaped body with conductive tree-shaped HCCs at micro- and nanoscale, and the results manifested that increasing the number of HCCs didn’t appear to necessarily improve the HCP of the disc.

However, as a matter of fact, the heat generation of practical electronic devices are usually non-uniformly distributed due to the working conditions and operating environment. In this context, considering the non-uniform heat generating phenomenon makes the research outcomes closer to real life problems in the field of electronics cooling, which is of great pragmatic significance. As mentioned above, most of the constructal researches in recent years were apparently carried out under the precondition of uniform heat generation, which was incompatible with the actual situation. Cetkin and Oliani [55] investigated rectangular constructs with non-uniform heat generation (NUHG), and optimized the shapes of HCCs on account of the minimum MTD. Besides, they analyzed the influences of HCC locations and shapes on the hot spot temperature in the premise of linear and local heat generation, respectively. Feng et al. [56,57] constructed the NUHG rectangular bodies inserted with constant and variable cross-sectional HCCs, respectively, and numerically obtained the optimal constructs with the criteria of minimum MTD [56] and minimum entransy dissipation rate [57], respectively. You et al. [58] performed constructal optimization of a NUHG triangular body, and theoretically and numerically optimized the first order constructs with constant and variable cross-sectional HCCs, respectively. The corresponding results showed that the minimum MTD of the constructs with variable cross-sectional HCCs was decreased by 12.6% compared with the one with constant cross-sectional HCCs, which thereby meant that the HCP could be further improved by adopting the variable cross-sectional HCCs structure in this regard.

Among the heat conduction constructal investigations, the disc model with NUHG is rarely considered. On the basis of [28,46,55,56,57,58], a conduction model in the radial-pattern disc considering NUHG phenomenon will be established in this paper. Constructal optimizations of the radial-pattern disc with constant cross-sectional HCCs will be carried out analytically and numerically, and the minimum MTDs derived by the two methods will be compared. In order to quest for a better heat transfer performance, discarding the constraint that the width of HCC is constant, variable cross-sectional HCC architecture [28] will be further utilized in the radial-pattern disc model in the meantime. Thereafter, the influences of heat generating distribution and HCC shapes on the optimal results will be analyzed.

## 2. Constructal Optimization of a Radial-Pattern Disc with Analytical Solution

**Figure 1:**
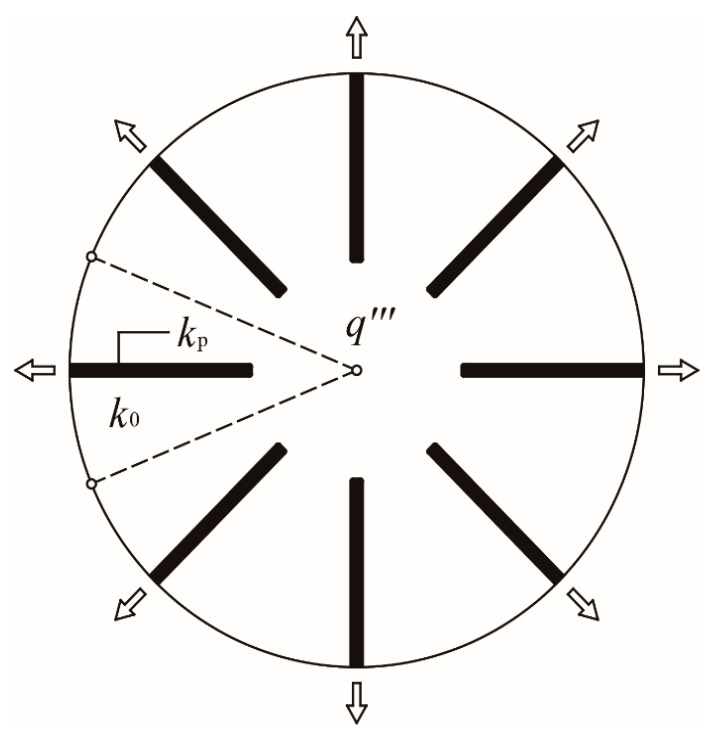
Heat conductive model of a radial-pattern disc with nonuniform heat generating.

A generic heat conduction model of a radial-pattern disc is schematically shown in Figure 1. As shown in this figure, the radius of the disc is R, and the heat generating rate (HGR) in the disc (thermal conductivity $k_{0}$) is $q^{\prime \prime \prime }={q_{0}}^{\prime \prime \prime }\cdot f(r)$, where ${q_{0}}^{\prime \prime \prime }$ refers to the heat generating constant and f(r) refers to the HGR function. The heat generated in the radial-pattern disc converges into N HCCs (length L and width D) which are uniformly distributed in the circumferential direction. By this means, the heat flows out of the disc-shaped body from the ends of HCCs which are situated on the rim of the disc.

Given that the total amount of high conductivity material (thermal conductivity $k_{p}$) is fixed, the HCCs could extend radially from the perimeter to the center of the disc. In this manner, the area fraction ϕ is defined as the ratio of $k_{p}$ area to the disc area:

(1) $$\phi =DLN/(\pi R^{2})$$

**Figure 2:**
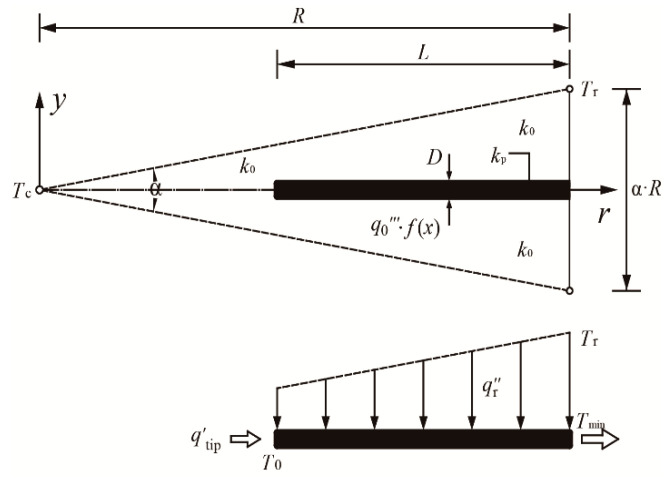
Simplified triangular element with nonuniform heat generation and constant cross-sectional high conductivity channel.

According to the distribution of the HGR and HCCs, the radial-pattern disc can be divided into N (=2π/α) identical sectorial elements (i.e., the number of HCCs in the disc), where α is the apex angle of each sectorial element. When the apex angle α (=2π/N) is specified, the HCP of the radial-pattern disc can vary with the length ratio λ (=L/D) of HCCs. Supposing that the apex angle α is sufficiently small, each sectorial element can be approximately viewed as an isosceles triangle, whose base and height are α⋅R and R, respectively. In this way, as shown in Figure 2, the triangular element (TE) consists of two parts, i.e., a trapezoid area which contains an HCC and a wedge-shaped area near the apex of the TE.

The total heat current generated in the sectorial area per unit of thickness is:

(2) $$q^{\prime }={q_{0}}^{\prime \prime \prime }\alpha R^{2}/2$$

Clearly, the MTD in the TE appears to be $T_{\max}-T_{\min}$, where $T_{\max}$ denotes the hot spot temperature and $T_{\min}$ denotes the HCC temperature on the rim. With the exception of the end of HCC ($T_{\min}$), the arc boundary is the same adiabatic condition as the radial boundaries, which are sketched with dashed lines. In the case that the TE’s apex angle α is sufficiently small, the hot spot may occur at the center of the disc (temperature $T_{c}$), or may probably occur on the TE’s base far from the HCC (temperature $T_{r}$). In this manner, the hot spot temperature can be inspected as:

(3) $$T_{\max}=\max(T_{c},\;T_{r})$$

The dimensionless temperature difference in the TE is defined as:

(4) $$\Delta \tilde{T}=(T-T_{\min})/({q_{0}}^{\prime \prime \prime }R^{2}/k_{0})$$

Likewise, the structure parameters related to the TE are non-dimensionalized as:

(5) $$(\tilde{L},\;\tilde{D},\;\tilde{r})=(L,\;D,\;r)/R$$

In line with Ref. [57], assuming that the average HGR of the whole disc is a constant, the HGR decreases gradually along the radius, so that the HGR function f(r) is written as $f(r)=1+0.1p-0.15p\tilde{r}$, in which p is termed as the nonuniform heat generating (NUHG) coefficient.

The heat current $q_{tip}^{\prime }$ generated in the wedge-shaped area (radius R−L and apex angle α) is calculated as:

(6) $$q_{tip}^{\prime }={q_{0}}^{\prime \prime \prime }(10+p\tilde{L})/20\cdot \alpha {\left(R-L\right)}^{2}$$

Moreover, the heat flux ${q_{r}}^{\prime \prime }$ collected at the longitudinal direction of y axis is given as:

(7) $${q_{r}}^{\prime \prime }={q_{0}}^{\prime \prime \prime }(1+0.1p-0.15p\tilde{r})(\alpha r-D)$$

According to [46,57], in the context of ϕ<<1, $k_{0}<<k_{p}$ and sufficiently small α, the heat conduction direction in the $k_{p}$ material can be approximately viewed as parallel to the r axis, while that in the $k_{0}$ material can be viewed as parallel to the y axis. Based on the above hypotheses, the heat conduction differential equation in the $k_{p}$ material is:

(8) $$\frac{d}{dr}(-k_{p}D\frac{dT}{dr})={q_{r}}^{\prime \prime }$$

The corresponding boundary conditions are invoked as:

(9) $$-k_{p}D\frac{dT}{dr}=q_{tip}^{\prime },\;r=R-L$$

(10) $$T=T_{\min},\;r=R$$

By solving Equations (8)–(10), the dimensionless temperature difference along the HCC is:

(11) $$\frac{T_{0}-T_{\min}}{{q_{0}}^{\prime \prime \prime }R^{2}/k_{0}}=\frac{\lambda }{\tilde{k}}\{\alpha [\frac{p}{80}{\tilde{L}}^{3}+\frac{1}{2}+{\tilde{L}}^{2}(\frac{1}{6}-\frac{p}{30})+\tilde{L}(-\frac{1}{2}+\frac{1}{40}p)]+\tilde{D}\tilde{L}(-\frac{1}{2}-\frac{p}{20}\tilde{L}+\frac{p}{40})\}$$

where $\tilde{k}$ denotes the ratio of $k_{p}$ to $k_{0}$, and $T_{0}$ denotes the temperature of the HCC terminal near the center of the disc.

In addition, the heat conduction differential equation in the wedge-shaped area is:

(12) $$\frac{d}{dr}(-k_{0}\alpha \tilde{r}\frac{dT}{dr})=\alpha \tilde{r}{q_{0}}^{\prime \prime \prime }(1+0.1p-0.15p\tilde{r})$$

The corresponding boundary conditions are invoked as:

(13) $$-k_{0}\alpha r\frac{dT}{dr}=q_{tip}^{\prime },\;r=R-L$$

(14) $$T=T_{c},\;r=0$$

Integrating Equations (12)–(14) yields the dimensionless temperature difference between $T_{c}$ and $T_{0}$

(15) $$\frac{T_{c}-T_{0}}{{q_{0}}^{\prime \prime \prime }R^{2}/k_{0}}=\frac{1}{120}(30+2p\tilde{L}+p){\left(1-\tilde{L}\right)}^{2}$$

Combining Equations (11) and (15) leads to the dimensionless temperature difference between $T_{c}$ and $T_{\min}$. Therefore, the dimensionless temperature difference ${\tilde{T}}_{c}$ can be defined as:

(16) $$\begin{array}{l} {\tilde{T}}_{c}=(T_{c}-T_{\min})/({q_{0}}^{\prime \prime \prime }R^{2}/k_{0})\\ \;=\frac{\alpha }{2}\{\frac{\lambda }{\tilde{k}}(\frac{p}{40}{\tilde{L}}^{3}+\frac{5-p}{15}{\tilde{L}}^{2}+\frac{p-20}{20}\tilde{L}+1)+\frac{1}{\tilde{k}\alpha }[-1-\frac{p}{10}\tilde{L}+\\ \;+\frac{p}{40}]{\tilde{L}}^{2}+\frac{1}{\alpha }(\frac{30+p}{60}+\frac{p}{30}\tilde{L})(1+{\tilde{L}}^{2}-2\tilde{L})\} \end{array}$$

From Equation (1), $\tilde{L}$ can be inspected as:

(17) $$\tilde{L}={\left(\alpha /2\cdot \phi \lambda \right)}^{1/2}$$

In terms of the structure of the TE, the HCC is geometrically contained in it, which should meet the following constraint:

(18) D<α(R−L)

As discussed previously, another way to express Equation (18) is:

(19) $$[1+1/(\alpha \lambda )]\cdot {\left(\alpha /2\cdot \phi \lambda \right)}^{1/2}<1$$

From Equation (3), the temperature difference $T_{r}-T_{\min}$ should be compared with $T_{c}-T_{\min}$ to determine the MTD ($T_{\max}-T_{\min}$), i.e.:

(20) $$T_{\max}-T_{\min}=\max(T_{c}-T_{\min},\;T_{r}-T_{\min})$$

The heat conduction differential equation in the $k_{0}$ material of trapezoid area is:

(21) $$\frac{d^{2}T}{dy^{2}}+\frac{{q_{0}}^{\prime \prime \prime }(1+0.1p-0.15p\tilde{r})}{k_{0}}=0$$

The corresponding boundary conditions are invoked as:

(22) $$\frac{dT}{dy}=0,\;y=\alpha r/2$$

(23) $$T=T_{\min},\;y=0$$

Integrating Equations (21)–(23), the temperature difference $T_{r}-T_{\min}$ on the base of the TE can be quantified as:

(24) $$T_{r}-T_{\min}={q_{0}}^{\prime \prime \prime }(1-0.05p)\alpha^{2}R^{2}/(8k_{0})$$

The corresponding ${\tilde{T}}_{r}$ is also specified as:

(25) $${\tilde{T}}_{r}=(T_{r}-T_{\min})/({q_{0}}^{\prime \prime \prime }R^{2}/k_{0})=(1-0.05p)\alpha^{2}/8$$

Hence, the dimensionless MTD $\Delta \tilde{T}$ of the heat conduction disc is the larger one between ${\tilde{T}}_{c}$ and ${\tilde{T}}_{r}$, that is:

(26) $$\Delta \tilde{T}=\max({\tilde{T}}_{c},\;{\tilde{T}}_{r})$$

In view of Equation (16), under given conditions that the apex angle α, area ratio ϕ and conductivity ratio $\tilde{k}$ are fixed, ${\tilde{T}}_{c}$ can be described as a function of the dimensionless HCC length $\tilde{L}$. By this means, the optimal $\tilde{L}$ (${\tilde{L}}_{opt}$) can be derived by solving $\partial {\tilde{T}}_{c}/\partial (\tilde{L})=0$, which is implicitly given by:

(27) $$\begin{array}{l} \frac{1}{\tilde{k}\phi }[15p{\tilde{L}}_{opt}{}^{4}+(160-32p){\tilde{L}}_{opt}{}^{3}+(-360+18p){\tilde{L}}_{opt}{}^{2}+240{\tilde{L}}_{opt}]+\frac{1}{\tilde{k}}[-18p{\tilde{L}}_{opt}{}^{2}+\\ (-120+3p){\tilde{L}}_{opt}]+6\;[p{\tilde{L}}_{opt}{}^{2}+(10-p){\tilde{L}}_{opt}-10]=0 \end{array}$$

By Equations (16) and (17), ${\tilde{T}}_{c,\min}$ is depicted as:

(28) $$\begin{array}{ll} {\tilde{T}}_{c,\min}= & \frac{1}{2}\{\frac{1}{\tilde{k}\phi }[\frac{p}{20}{\tilde{L}}_{opt}{}^{5}+\frac{10-2p}{15}{\tilde{L}}_{opt}{}^{4}+\frac{p-20}{10}{\tilde{L}}_{opt}{}^{3}+2{\tilde{L}}_{opt}{}^{2}]+\frac{1}{\tilde{k}}[-\frac{p}{10}{\tilde{L}}_{opt}{}^{3}+\\ & \frac{p-40}{40}{\tilde{L}}_{opt}{}^{2}]+\frac{p}{30}{\tilde{L}}_{opt}{}^{3}+\frac{10-p}{20}{\tilde{L}}_{opt}{}^{2}-{\tilde{L}}_{opt}+\frac{30+p}{60}\} \end{array}$$

**Figure 3:**
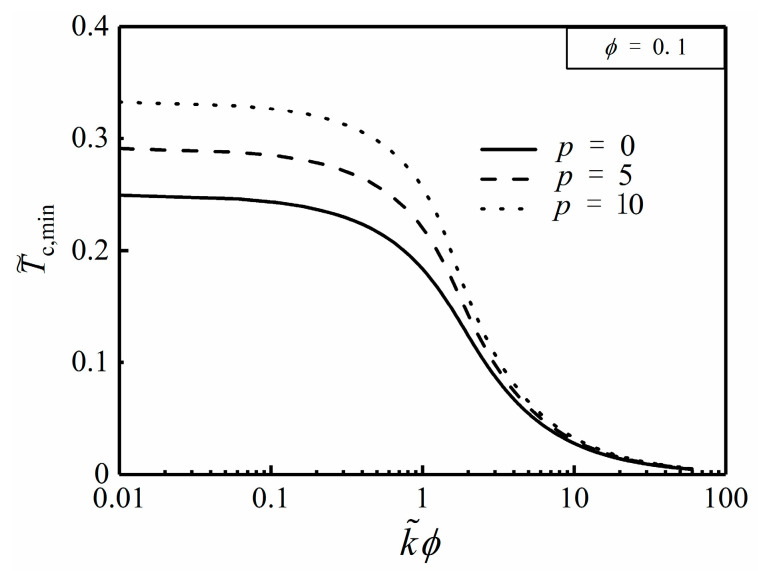
Effect of p on ${\tilde{T}}_{c,\min}$ versus $\tilde{k}\phi$.

**Figure 4:**
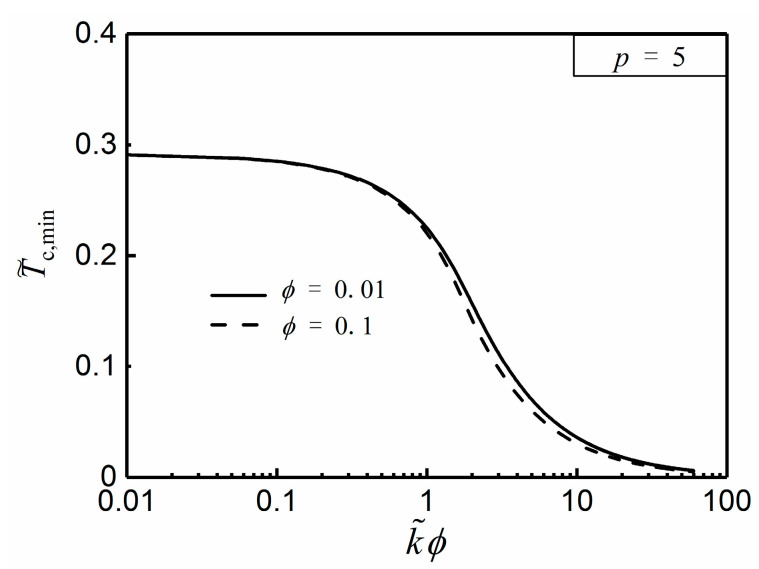
Effect of ϕ on ${\tilde{T}}_{c,\min}$ versus $\tilde{k}\phi$.

From Equation (27), the optimal HCC length (${\tilde{L}}_{opt}$) is definitely independent of α, but merely related to ϕ and $\tilde{k}$. Figure 3 and Figure 4 depict the effects of the NUHG coefficient p and area ratio ϕ on the relationship of ${\tilde{T}}_{c,\min}$ and $\tilde{k}\phi$, respectively. From Figure 3, one can see that ${\tilde{T}}_{c,\min}$ tends to be 0 when $\tilde{k}\phi >>10$, i.e., ${\tilde{T}}_{c,\min}$ is rather minor when $\tilde{k}$ is far larger than 1, so that the HCP can be improved by increasing the conductivity ratio in this regard. Additionally, ${\tilde{T}}_{c,\min}$ increases with the increase of p when $\tilde{k}\phi$ is given, and the HCP is weaken. From Figure 4, when $\tilde{k}\phi$ is far less than 1, ${\tilde{T}}_{c,\min}$ is equal to 0.291. By using Equation (25), α needs to be 1.762 when ${\tilde{T}}_{r,\min}=0.291$, but this could be seen as a breach of the prior assumption of sufficiently small apex angle α. Hence, one can highlight that the hot spot is always situated at the center of the disc without exception.

**Figure 5:**
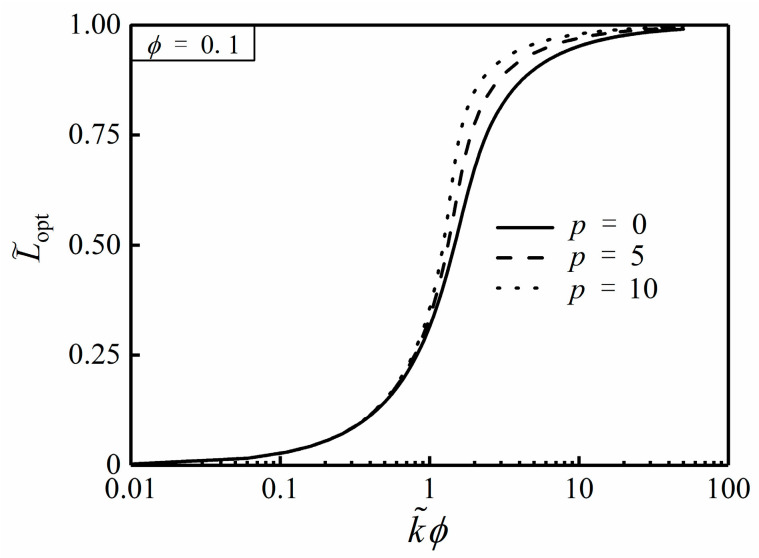
Effect of p on ${\tilde{L}}_{opt}$ versus $\tilde{k}\phi$.

**Figure 6:**
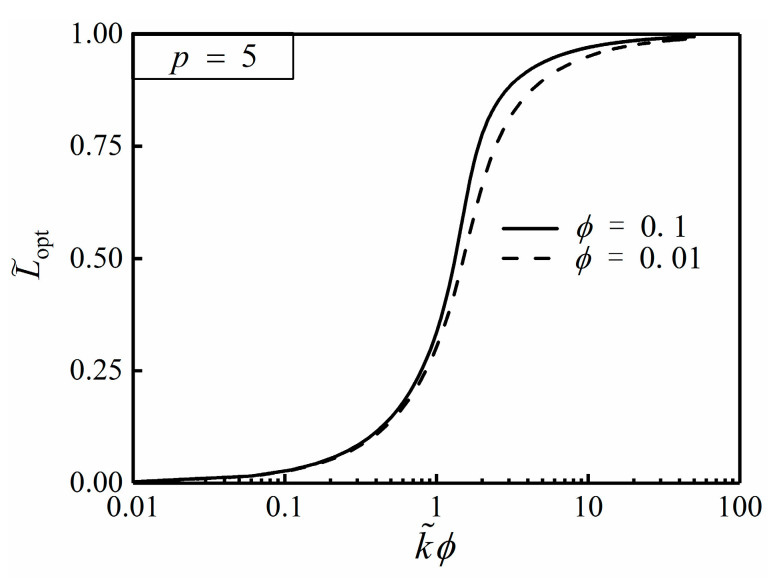
Effect of ϕ on ${\tilde{L}}_{opt}$ versus $\tilde{k}\phi$.

Figure 5 and Figure 6 depict the effects of the NUHG coefficient p and area ratio ϕ on the relationship of ${\tilde{L}}_{opt}$ and $\tilde{k}\phi$, respectively. From Figure 5, when p is initially specified, ${\tilde{L}}_{opt}$ increases in an S-shaped mode with the increase of $\tilde{k}\phi$, and obviously appears to have a quick change between 1 and 10. When $\tilde{k}\phi <<1$, ${\tilde{L}}_{opt}$ tends to be 0, and the high conductivity material is nearly stacked on the rim of the disc in this condition. When $\tilde{k}\phi >>10$, ${\tilde{L}}_{opt}$ tends to be 1, and the HCC almost extends to the center of the disc. When $\tilde{k}\phi$ is given, ${\tilde{L}}_{opt}$ increases as p increases. In this case, the larger the HGR near the center of the disc is, the longer the HCC is in order to deliver the generated heat and reduce the temperature difference efficiently. From Figure 6, when $\tilde{k}\phi$ is specified, ${\tilde{L}}_{opt}$ increases as ϕ increases. This is because the HCC needs to be longer to deliver more heat current near the center of the disc.

## 3. Constructal Optimization of a Radial-Pattern Disc with Numerical Solution

When the presuppositions of ϕ<<1, $k_{0}<<k_{p}$ and small α are untenable, the actual heat conduction in $k_{0}$ and $k_{p}$ materials are both no longer a simple one-dimensional one, and Equations (8), (12) and (21) are invalidated in this manner. This problem always exists in analytical solution, and one can solve it by using higher dimensional model with numerical solution. As shown in Figure 2, the two-dimensional heat conduction differential equations in the $k_{0}$ and $k_{p}$ materials are, respectively, given as:

(29) $$\frac{\partial^{2}T}{\partial r^{2}}+\frac{\partial^{2}T}{\partial y^{2}}+\frac{{q_{0}}^{\prime \prime \prime }\cdot f(r)}{k_{0}}=0$$

(30) $$\frac{\partial^{2}T}{\partial r^{2}}+\frac{\partial^{2}T}{\partial y^{2}}=0$$

With a view of the symmetric geometry of the TE, there appears to be sufficient to merely investigate the area of y≥0. In this regard, the requisite boundary conditions are:

(31) $$T=T_{\min}\;r=R,\;0<y\leq D/2$$

(32) ∂T/∂r=0  r=R,  D/2<y≤αR/2

(33) $$\partial T/\partial n=0\;\left\{ \begin{array}{l} 0<r\leq R,\;y=0\\ 0\leq r<R,\;y=\alpha r/2 \end{array}\right.$$

where n is termed as the normal vector to the corresponding border.

For simplification, it is assumed that there exists no thermal contact resistance at the interface between the $k_{0}$ and $k_{p}$ materials, so that the heat current continuity equation is:

(34) $$k_{0}{\left(\partial T/\partial n\right)}_{\mathrm{Disc}}=k_{p}{\left(\partial T/\partial n\right)}_{\mathrm{HCC}}$$

The dimensionless temperature difference and geometry parameters are defined the same as those in Equations (4) and (5). From Equation (4), one can know that the dimensionless MTD $\Delta {\tilde{T}}_{0}$ is

(35) $$\Delta {\tilde{T}}_{0}=(T_{\max}-T_{\min})/({q_{0}}^{\prime \prime \prime }R^{2}/k_{0})$$

where $\Delta {\tilde{T}}_{0}$ is the dimensionless MTD of the whole disc.

The PDE toolbox of Matlab is invoked to solve Equations (29)–(34), in which the finite element method is adopted. During the processes of numerical calculations, the target area is sequentially refined until the relative error of MTDs between $\Delta {\tilde{T}}_{0}{}^{j}$ and $\Delta {\tilde{T}}_{0}{}^{j-1}$ obtained from the jth and j−1th refinements satisfies:

(36) $$\left|(\Delta {\tilde{T}}_{0}{}^{j}-\Delta {\tilde{T}}_{0}{}^{j-1})/\Delta {\tilde{T}}_{0}{}^{j-1}\right|<0.005$$

According to Equations (16) and (17), it clearly indicates that when the conductivity ratio $\tilde{k}$, area ratio ϕ and HCC number N (=2π/α) are specified, the dimensionless MTD $\Delta {\tilde{T}}_{0}$ of the disc is only a function of the length ratio λ (=L/D). As a result, by taking the NUHG into consideration, the constructal optimization of the disc with constant and variable cross-sectional HCCs can be carried out with λ as the variable, respectively.

### 3.1. Constant Cross-Sectional HCCs

**Figure 7:**
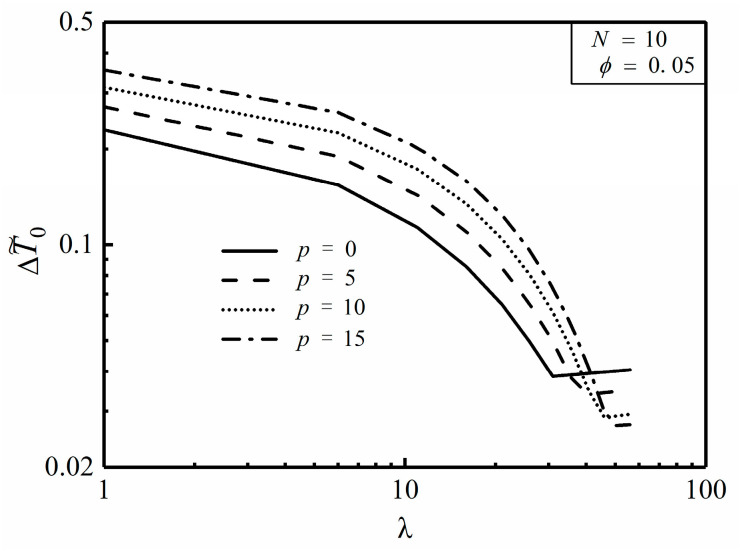
Effect of p on $\Delta {\tilde{T}}_{0}$ versus λ.

The constant cross-sectional HCCs model is the same as that shown in Figure 1 and Figure 2, and one can further optimize its HCP with using numerical solution as follows: Figure 7 depicts the effect of the NUHG coefficient p on the relationship of the dimensionless MTD $\Delta {\tilde{T}}_{0}$ and length ratio λ. It is easy to see from Figure 7 that when N, ϕ, $\tilde{k}$ and p are already given, there appears to be an optimal λ ($\lambda_{opt}$) leading to the minimum $\Delta {\tilde{T}}_{0}$ ($\Delta {\tilde{T}}_{0,\min}$), which means that choosing an appropriate λ can effectively reduce the MTD and improve the HCP to some extent. Furthermore, $\lambda_{opt}$ increases with the increase of p, in this case that the HCC needs to protrude into the center of the disc where the heat is relatively concentrated, which aims to reduce the MTD. On the one hand, the nonuniform heat generating model can be processed back to the model mentioned in [46] in the condition of p=0. On the other hand, when p=10, $\tilde{k}=5000$, ϕ=0.01 and N=100, $\Delta {\tilde{T}}_{0}$ is equal to 0.2764 by using the finite element method, and comparatively $\Delta {\tilde{T}}_{0}$ is equal to 0.2711 by using the analytical method in Equation (16). These preconditions mentioned above mainly meet the foundations of Equations (8), (12) and (21). Clearly, if given these prerequisites, the two-dimensional model can be simplified as a one-dimensional one. The relative error between these two results is merely 1.9%, which in the meantime endorses the validity of analytical method under the given assumptions. Besides, in case of p=10, $\tilde{k}=5$, ϕ=0.1 and N=25, $\Delta {\tilde{T}}_{0}$ is equal to 0.2686 and 0.3868 obtained from the finite element method and the analytical method, respectively, and the deviation between the two is severely 30.6%. In the context, the given conditions violate the establishing conditions of the analytical method, which means that one can accordingly choose a better way to calculate the MTD of the disc based on the given conditions.

**Figure 8:**
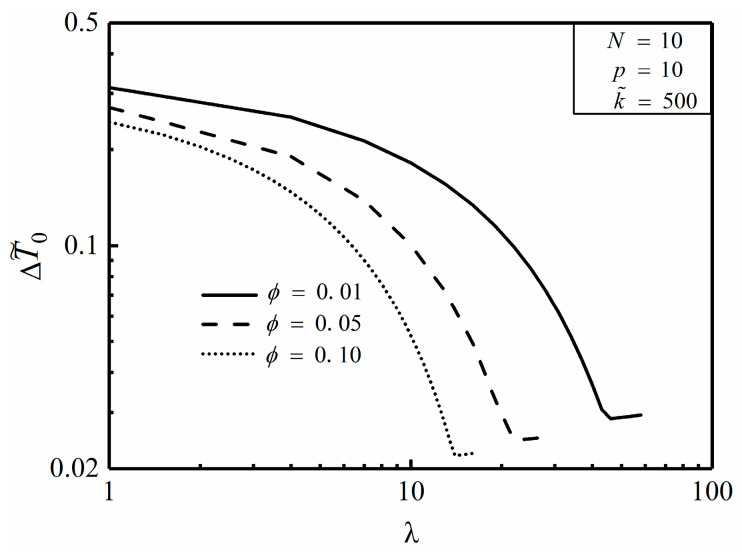
Effect of ϕ on $\Delta {\tilde{T}}_{0}$ versus λ.

**Figure 9:**
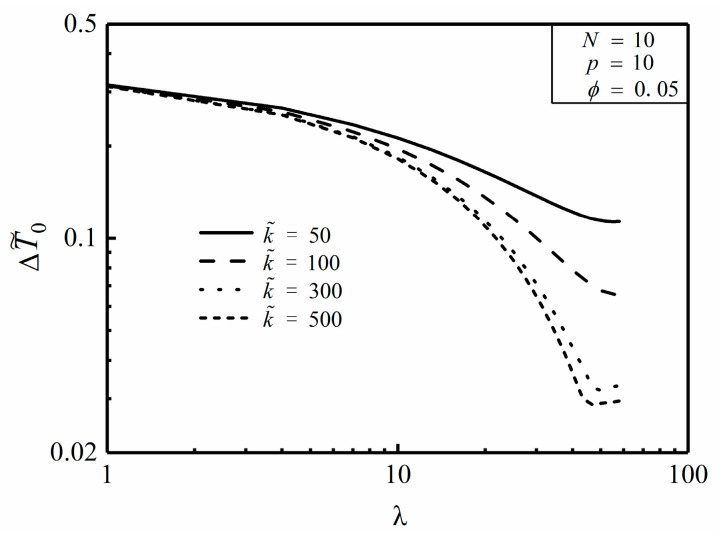
Effect of $\tilde{k}$ on $\Delta {\tilde{T}}_{0}$ versus λ.

Figure 8 and Figure 9 depict the effects of area ratio ϕ and conductivity ratio $\tilde{k}$ on the relationship of the dimensionless MTD $\Delta {\tilde{T}}_{0}$ and length ratio λ. It is easy to see from Figure 8 that when N, $\tilde{k}$ and p are already given, the minimum dimensionless MTD $\Delta {\tilde{T}}_{0,\min}$ decreases with the increase of ϕ as well as the corresponding $\lambda_{opt}$ does. This indicates that whereas more high conductivity material is available, the HCC seems to be thicker and shorter for a better HCP. From Figure 9, when N, ϕ and p are already given, both $\Delta {\tilde{T}}_{0,\min}$ and $\lambda_{opt}$ decrease with the increase of $\tilde{k}$, which means that the HCP can be improved by increasing the conductivity ratio.

**Figure 10:**
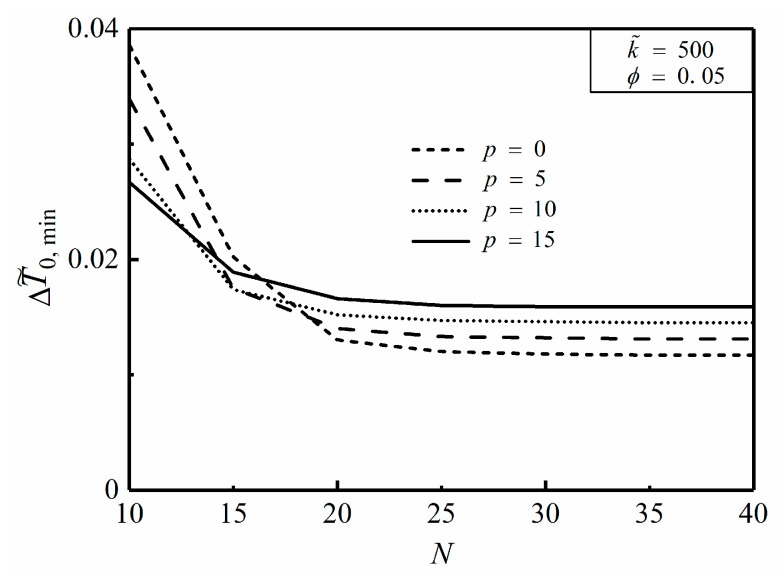
Effect of p on $\Delta {\tilde{T}}_{0,\min}$ versus N.

Figure 10 depicts the effect of the NUHG coefficient (p) on the relationship of the minimum dimensionless MTD $\Delta {\tilde{T}}_{0,\min}$ and HCC number N. From Figure 10, when p is equal to 0 and 15, $\Delta {\tilde{T}}_{0,\min}$ tends to be 0.0117 and 0.0159, respectively, with a large value of N. Apparently, the MTD can be reduced by increasing the HCC number on condition that N varies from 10 to 25. When p is equal to 10, $\Delta {\tilde{T}}_{0,\min}$ of the disc with 25 HCCs is specifically reduced by 48.8% compared to that with 10 HCCs. Instead, $\Delta {\tilde{T}}_{0,\min}$ remains constant when N varies from 25 to 40. Whereupon, one can speculate that it is costly to further increase the number of HCCs when N>25, and the HCP is not improved to some extent in this case.

### 3.2. Variable Cross-Sectional HCCs

**Figure 11:**
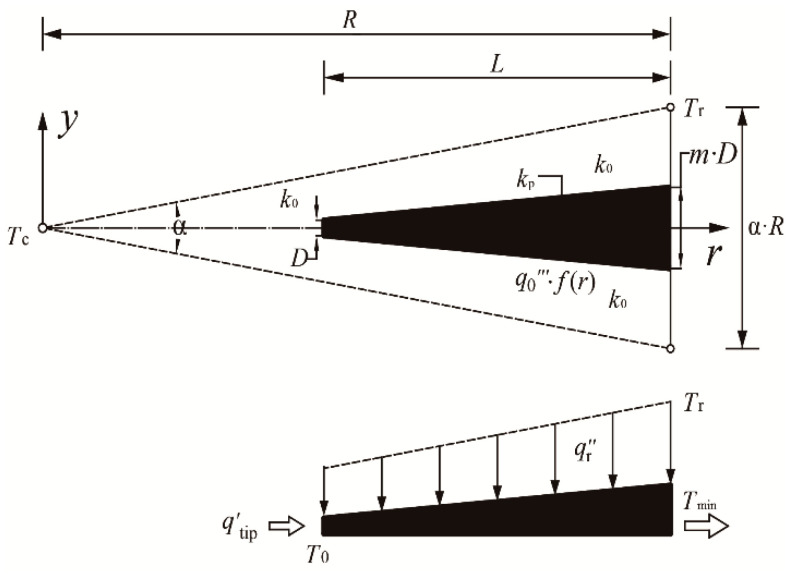
Simplified triangular element with nonuniform heat generating and variable cross-sectional high conductivity channel.

In order to further improve the HCP of the disc, the model of a TE with variable cross-sectional [28,29] HCCs is established as shown in Figure 11. The minimum and maximum widths of the HCCs are D and m⋅D, respectively, where m is termed as the width ratio. The area ratio ϕ of $k_{p}$ material is:

(37) $$\phi =DLN\cdot (m+1)/(2\pi R^{2})$$

Similarly, the dimensionless $\tilde{L}$ is:

(38) $$\tilde{L}={\left[\alpha /(m+1)\cdot \lambda \phi \right]}^{1/2}$$

When the width ratio m is specified, constructal optimizations of the disc with variable cross-sectional HCCs can be carried out similar to that in Section 3.1. By invoking the PDE toolbox, one can numerically obtain the MTD in the heat generating area according to Equations (37) and (38) and the numerical method given in Section 3.1.

**Figure 12:**
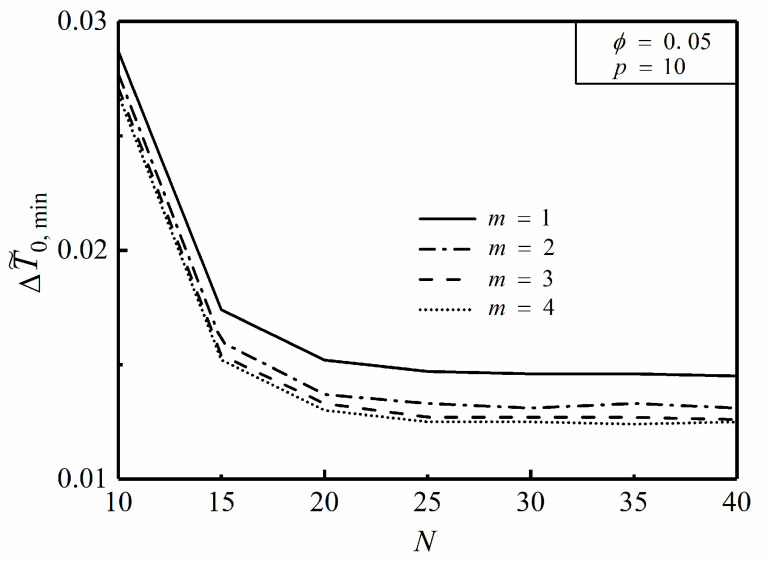
Effect of m on $\Delta {\tilde{T}}_{0,\min}$ versus N.

**Figure 13:**
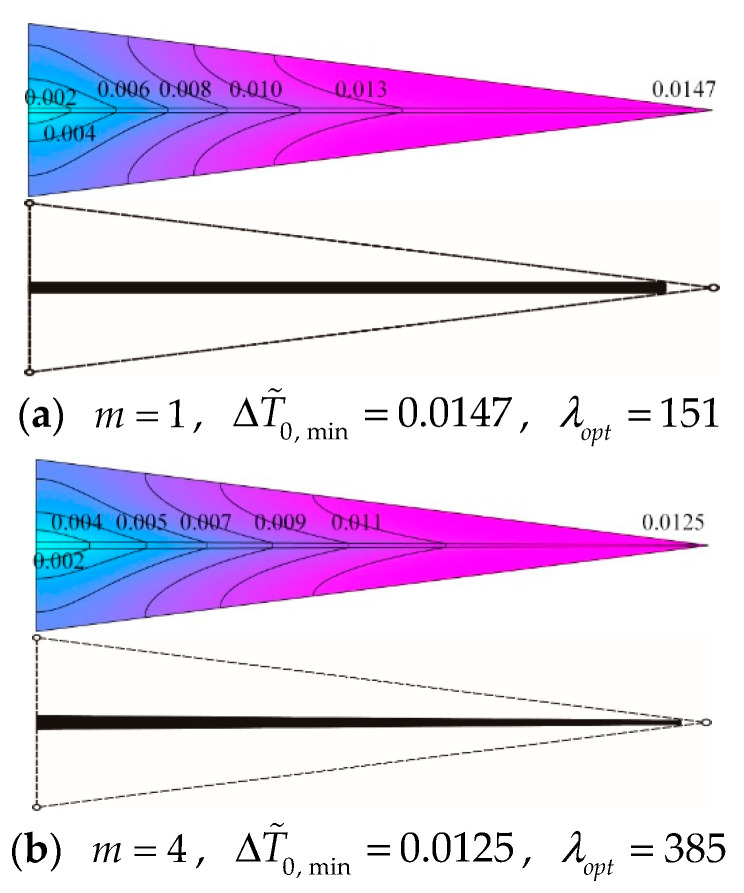
Optimal construct comparison of triangular elements with different high conductivity channels: (**a**) constant cross-section, (**b**) variable cross-section.

Figure 12 depicts the effect of the width ratio m on the relationship of the minimum dimensionless MTD $\Delta {\tilde{T}}_{0,\min}$ and HCC number N with ϕ=0.05 and p=10. From Figure 12, it is clear that $\Delta {\tilde{T}}_{0,\min}$ first decreases and then remains a constant with the increase of N. Otherwise, when N is given, $\Delta {\tilde{T}}_{0,\min}$ gradually decreases as m increases. According to [46], when the disc is uniformly heat generating and inserted with constant cross-sectional HCCs, a.k.a. p=0, $\Delta {\tilde{T}}_{0,\min}$ is equal to 0.0107 in terms of $\tilde{k}=500$, ϕ=0.1 and N=20. While applying the variable cross-sectional structure in the disc under the same constraint conditions, $\Delta {\tilde{T}}_{0,\min}$ is equal to 0.0097 which is reduced by 9.3%. In these two cases, the optimal constructs of HCCs both have no geometrical connections with the center of the disc. From Figure 12, one can see that as the disc consists of 15 TEs and the heat is nonuniformly generated, $\Delta {\tilde{T}}_{0,\min}$ is equal to 0.0174 in case of m=1 while $\Delta {\tilde{T}}_{0,\min}$ is equal to 0.0152 in case of m=4, that is, $\Delta {\tilde{T}}_{0,\min}$ is decreased by 12.6% when m changes from 1 to 4. In these two nonuniform heat generating cases, the optimal constructs of HCCs likewise have no geometrical connections with the center. As revealed in Figure 13a,b the isothermal lines are labelled with corresponding dimensionless temperature differences. In the premise of ϕ=0.05, p=10 and N=25, for the disc with constant cross-sectional HCCs (i.e., m=1), the optimal length ratio ($\lambda_{opt}$) turns out to be 151 resulting in $\Delta {\tilde{T}}_{0,\min}$, which is equal to 0.0147. Besides, for the disc with variable cross-sectional HCCs (i.e., m=4), $\lambda_{opt}$ turns out to be 385 resulting in $\Delta {\tilde{T}}_{0,\min}$, which is equal to 0.0125. It can be seen that $\Delta {\tilde{T}}_{0,\min}$ of the disc with m=4 is decreased by 15.0% compared to that with m=1. When the disc is nonuniformly heat generating, heat is relatively concentrated near the heat sink, i.e., the ends of HCCs situated on the perimeter of the disc. It is beneficial to place more high conductivity material near the heat sink so as to efficiently transfer the heat current. Therefore, the geometry of variable cross-sectional HCCs takes advantage of distributing more high conductivity material near the region where heat is more concentrated, which can be applied in the constructal design of radial-pattern disc to improve its HCP.

## 4. Conclusions

With the high demand to efficient cooling systems for heat generating area, the heat conduction model of a radial-pattern disc considering the NUHG is established in this paper, which is closer to real problems in the field of electronics cooling. Constructal optimizations of the discs with constant and variable cross-sectional HCCs are implemented, and their MTDs are minimized. The influences of the NUHG coefficient p, HCC number N and conductivity ratio $\tilde{k}$ on the optimal results are analyzed. The HCP comparisons obtained by analytical and numerical solutions as well as constant and variable cross-sectional HCCs are carried out. The results are given as follows:

(1) For the disc with constant cross-sectional HCCs, the optimal length ratio $\lambda_{opt}$ increases with the increase of p, in this case that the HCCs need to protrude into the center of the disc where the heat is relatively concentrated. The deviation of the optimal constructs obtained from analytical method and finite element method is comparatively slight. When p is equal to 10, $\Delta {\tilde{T}}_{0,\min}$ of the disc with 25 HCCs is specifically reduced by 48.8% compared to that with 10 HCCs. As a result, the HCP of the disc can be efficiently improved by properly increasing the number of HCCs. In addition, the approach of increasing $\tilde{k}$ can also contribute to reduce the MTD and improve the HCP of the disc.
(2) For the disc with variable cross-sectional HCCs, the $\Delta {\tilde{T}}_{0,\min}$ of the disc in case of m=4 is decreased by 15.0% compared to that of m=1 in the premise of ϕ=0.05, p=10 and N=25. Therefore, the geometry of variable cross-sectional HCCs takes advantage of distributing more high conductivity material near the region where heat is more concentrated, which can be applied in the constructal design of radial-pattern disc to improve its HCP.

In this paper, the heat conduction problem of the radial pattern disc with a single high conductivity channel arranged in each element is studied. In the following research, the radial-pattern disc with more complex multistage tree-shaped high conductivity channels will be investigated wishing to further improve the heat transfer performance. Furthermore, the effects of the location where the heat sink is situated on the heat conduction performance can be studied. Additionally, the percentage of the surface/volume in which the temperature is above a certain value is worth taking into consideration to improve the existing work.

## Nomenclatures

D	Width [m]
f(r)	Heat generating rate function
$k_{0}$	Thermal conductivity of heat generating area [W/(m·K)]
$k_{p}$	Thermal conductivity of high conductivity material [W/(m·K)]
L	Length [m]
m	Width ratio
N	Number of high conductivity channels
p	Nonuniform heat generating coefficient
${q_{0}}^{\prime \prime \prime }$	Heat generating constant in per unit volume [W/m^3^]
${q_{r}}^{\prime \prime }$	Heat current collected at the longitudinal direction of y axis [W/m^2^]
$q_{tip}^{\prime }$	Heat current $q_{tip}^{\prime }$ generated in the wedge-shaped area [W/m]
R	Radius [m]
r	Horizontal axis
T	Temperature [K]
y	Vertical axis
Greek symbols	
α	Apex angle of each sectorial element
λ	Length ratio L/D
ϕ	Area ratio of high conductivity material area to whole disc area
Subscripts	
*c*	Center of disc
*max*	Maximum
*min*	Minimum
*opt*	Optimum
*r*	Rim of disc
superscript	
~	nondimensionalized

## Abbreviations

The following abbreviations are used in this manuscript:

HCC	High conductivity channel
HCP	Heat conduction performance
HGR	Heat generating rate
MTD	Maximum temperature difference
NUHG	Nonuniform heat generation
